# Interplay between the Redox System and Renal Tubular Transport

**DOI:** 10.3390/antiox13101156

**Published:** 2024-09-24

**Authors:** Xiao-Lan Wang, Lianjian Li, Xianfang Meng

**Affiliations:** 1 Department of Nephrology, Union Hospital, Tongji Medical College, Huazhong University of Science and Technology, Wuhan 430022, China; xiaolan_wang@hust.edu.cn; 2 Department of Vascular Surgery, Hubei Provincial Hospital of Traditional Chinese Medicine, Affiliated Hospital of Hubei University of Traditional Chinese Medicine, Hubei Academy of Chinese Medicine, Wuhan 430061, China; lilianjian1115@sina.com; 3 Department of Neurobiology, Institute of Brain Research, School of Basic Medical Sciences, Tongji Medical College, Huazhong University of Science and Technology, Wuhan 430030, China

**Keywords:** redox system, metabolism, tubular transport, kidney

## Abstract

The kidney plays a critical role in maintaining the homeostasis of body fluid by filtration of metabolic wastes and reabsorption of nutrients. Due to the overload, a vast of energy is required through aerobic metabolism, which inevitably leads to the generation of reactive oxygen species (ROS) in the kidney. Under unstressed conditions, ROS are counteracted by antioxidant systems and maintained at low levels, which are involved in signal transduction and physiological processes. Accumulating evidence indicates that the reduction–oxidation (redox) system interacts with renal tubular transport. Redox imbalance or dysfunction of tubular transport leads to renal disease. Here, we discuss the ROS and antioxidant systems in the kidney and outline the metabolic dysfunction that is a common feature of renal disease. Importantly, we describe the key molecules involved in renal tubular transport and their relationship to the redox system and, finally, summarize the impact of their dysregulation on the pathogenesis and progression of acute and chronic kidney disease.

## 1. Introduction

The kidneys receive approximately 1/4–1/5 of the cardiac output, which is a high flow of oxygenated blood. The glomerular filtration rate (GFR) is approximately 120 mL per minute (180 L per day), while the average urine output is only about 1.5 L per day [1]. Most of the water and nutrients, such as glucose, amino acids, and electrolytes, are reabsorbed by the renal tubular cells, which require a high energy supply [2]. Additionally, the kidneys play an important role in removing metabolic wastes and toxins, maintaining electrolyte and fluid balance, and controlling pH homeostasis [3]. It has been estimated that the metabolic rate in the human kidneys is >400 kcal/kg tissue/day [4]. A variety of nutrients, such as glucose, fatty acids, lactate, and glutamine, are used as fuel to produce adenosine triphosphate (ATP) in the kidney. These biological processes are mediated by an array of membrane transporters, such as sodium-glucose co-transporter (SGLT), Na^+^-K^+^-ATPase, cluster of differentiation 36 (CD36), and others.

During aerobic metabolic processes such as oxidative phosphorylation (OXPHOS), reactive oxygen species (ROS) are generated by mitochondria as metabolic by-products. Under physiological conditions, ROS are counterbalanced by an antioxidant defense network that modulates ROS levels to enable their physiological roles in cellular signaling pathways and biological processes, including cell metabolism, differentiation, proliferation, angiogenesis, salt and fluid homeostasis, immune response, autophagy, and phosphorylation/dephosphorylation [5,6,7,8]. In particular, the kidneys are potentially exposed to high concentrations of oxidants and reactive electrophiles and are highly dependent on an adequate supply of antioxidants to maintain normal function. Reduction–oxidation (redox) imbalance and excess of ROS have detrimental effects on cell structure and function and contribute to intracellular oxidative stress, which results in inflammation, fibrosis, apoptosis, and cell damage in the kidney. Thus, loss of cellular redox homeostasis is a risk factor for the onset and progression of a variety of renal diseases.

Increasing evidence shows that redox signaling regulates tubular transport functions [9,10,11,12,13]. In turn, the transport of sugars, fatty acids, amino acids, ions, and other substances by membrane transporters can trigger a series of physiological responses that affect the redox system in the kidney [14,15]. In this review, we provide a brief overview of the redox system, renal metabolism, and the effects of metabolism on ROS production; summarize the interplay between tubular transport and the redox system; as well as describe how the redox imbalance contributes to renal disease.

## 2. Redox System in the Kidney

The kidney is a high energy-demanding organ that relies on the mitochondria to produce large amounts of ATP. During the process of oxidative phosphorylation (OXPHOS), the electron transport chain (ETC) shuttles electrons to molecular oxygen, and the production of oxygen-derived free radicals (also known as reactive oxygen species, ROS) is inevitable. Apart from mitochondria, nicotinamide adenine dinucleotide phosphate oxidases (NOX), peroxisomes, endoplasmic reticulum (ER), xanthine oxidase (XO), lipoxygenase, nitric oxide synthase (NOS), cyclooxygenases (COX), and cytochrome P450 monooxygenase are also sources of ROS in mammalian cells [16]. In the kidney, mitochondria and the NOX family are the major sources of endogenous ROS. Moreover, ROS are also induced by the formation of protein disulfide bridges or oxidase activity such as the induction of foreign substances, microbial invasion, and cytokines [6,17]. Moreover, exposure of cells and organisms to environmental oxidants such as ozone, nitrogen dioxide, and radiation also promotes ROS generation [18]. ROS include the superoxide anion (O_2_•^−^), hydrogen peroxide (H_2_O_2_), and hydroxyl radicals (OH•), etc. Additionally, the oxidant system includes numerous compounds containing both nitrogen and oxygen (called reactive nitrogen species, RNS), such as nitric oxide (NO), peroxynitrite (ONOO^−^), nitrotyrosine, and nitrosothiols [19]. ROS/RNS play a critical role in cell signaling pathways such as Nuclear Factor kappa B (NF-κB), mitogen-activated protein kinases (MAPKs), phosphatidylinositol-3-kinase (PI3K)-Akt, and the KEAP1-NRF2-ARE (Kelch-like ECH-Associating protein 1-nuclear factor erythroid 2 related factor 2-antioxidant response element) signaling pathways [20,21]. ROS can also reversibly oxidize redox-sensitive cysteine residues on target proteins and exert long-term cellular effects via epigenetic modifications [22,23].

The dynamic balance between intracellular oxidants and antioxidants is maintained by redox regulation. Redox reactions transfer electrons from reduced donor molecules to acceptor molecules. The major antioxidant system can be divided into enzymatic and non-enzymatic systems. The enzymatic system includes superoxide dismutases (SODs), catalase, glutathione peroxidase (GPx), glutathione reductase (GR), glutathione S-transferase (GST), peroxiredoxin (PRX), and thioredoxin (TRX) [6,24,25]. The non-enzymatic system consists of vitamin C, vitamin E, carotenoids, flavonoids, and glutathione (reduced glutathione or GSH) [24]. GSH is the major redox couple in cells and has been identified as a marker of the redox status in many diseases. The tripeptide GSH is synthesized from glutamate, cysteine, and glycine and is modulated by cysteine availability and GSH/GSSG feedback inhibition [26]. The GSH pool is tightly regulated and is linked to glucose oxidation via the pentose phosphate pathway (PPP), which provides the reducing power nicotinamide adenine dinucleotide phosphate (NADPH) to maintain GSH in a reduced state. GPx and GST perform the detoxification reactions through GSH, converting GSH to oxidized glutathione (GSSG); GR then reduces GSSG to GSH at the expense of NADPH, restoring the cellular GSH pool [27]. Nicotinamide adenine dinucleotide (NADH) acts as an antioxidant in both enzymatic and non-enzymatic systems, the best known role of NADH being in energy metabolism [28]. Similarly, NADPH, which is generated by NADH oxidation, is involved in cellular antioxidant defenses and is crucial for the recycling of GSH and thioredoxin [29].

In addition, GSH and associated enzymes are regulated by Nrf2—a master regulator of redox balance in the cellular cytoprotective response [24]. Under quiescent conditions, Nrf2 transcriptional activity is inhibited by its major inhibitor, Keap1, which acts as a redox sensor and targets Nrf2 for ubiquitination and subsequent proteasomal degradation [30]. However, under oxidative insults, Nrf2 is released from Keap1 and acts as a master transcription factor to induce the expression of genes with ARE to promote the antioxidant response process [31,32,33]. Nrf2 is expressed throughout the kidney and upregulates target genes such as NAD(P)H quinone dehydrogenase 1 (NQO1), glutathione S-transferases (GSTs), catalase (CAT), and heme oxygenase 1 (HMOX), which play critical roles in mitigating oxidative stress, participating in detoxification pathways and increasing glutathione synthesis [34,35,36,37].

In homeostasis, ROS/RNS are produced and maintained at low levels, and antioxidant systems counteract the damaging effects of ROS and RNS in the kidney [13,36,37,38]. However, due to the high oxygen and energy consumption, ROS formation is evident in the kidney, predominantly in the renal cortex, even under physiological conditions [38]. As hyperfiltration is a hallmark of the diabetic kidney, the energy demands of the proximal tubule are greatly increased [39]. Increased production of mitochondrial ROS has been reported in the early stages of DKD and is considered to be one of the primary factors causing diabetic tubular injury [40,41]. In CKD patients or animal models, ROS generation is more significant compared with controls [24,42,43]. Increased accumulation of ROS in the proximal tubules is a major contributor to renal pathology. When the oxidants are produced in large amounts, and the balance between ROS/RNS production and antioxidant defense is disturbed, oxidative stress occurs. Oxidative stress damages cellular components and is commonly observed in several renal diseases, such as diabetic kidney disease (DKD), acute kidney injury (AKI), and chronic kidney disease (CKD) [6,24,44,45,46].

## 3. Renal Metabolic Dysfunction in Pathological Conditions

The kidney is one of the most energy-demanding organs, requiring abundant ATP, most of which is generated by OXPHOS in the mitochondria [47]. Because of its high oxygen consumption, the kidney is more sensitive to changes in oxygen levels than other cell types [48]. Mitochondrial density in the kidney is one of the highest in the body to adapt to high energy requirements [49]. In particular, mitochondria are highly dynamic organelles, and their shape, distribution, and size are maintained by fission and fusion processes, which are closely linked to the kidney function [50,51]. However, mitochondrial defect leads to an energy deficit and cell death and affects cellular calcium levels and redox status [52]. Defective mitochondria are unable to maintain the proton gradient across the inner mitochondrial membrane and are the main source of ROS in cells [49,53]. In addition, NADPH oxidases (NOX) located in the mitochondria or at the plasma membrane also generate ROS [54]. Mitochondrial dysfunction has been regarded as a common feature and occurs early in a number of renal diseases, such as AKI and DKD [55,56]. Additionally, accumulating data suggest a strong association between the development and progression of renal diseases and mitochondrial dysfunction [4,57,58,59].

It has been revealed that >80% of the oxygen consumed by the kidneys is used by the proximal tubular cells to support the electrolyte and nutrient reabsorption [2]. Given this workload, non-esterified fatty acids (also known as free fatty acids, FFAs) are the main source of energy for renal tubular epithelial cells, which are then oxidized by fatty acid oxidation (FAO) and OXPHOS [52]. Total serum levels of FFAs have been reported to be elevated in patients with CKD compared with controls [60]. Specifically, saturated fatty acids and monounsaturated fatty acids are increased, whereas polyunsaturated fatty acids seem to be mostly decreased [61,62]. However, the polyunsaturated fatty acids (PUFA)-eicosapentaenoic acid (EPA) and docosahexaenoic acid (DHA) are renoprotective [63]. Lipid accumulation impairs mitochondrial dynamics and contributes to mitochondrial damage and increased ROS production [62]. In turn, dysfunction of mitochondrial oxidation and increased ROS reduce mitochondrial lipid utilization, leading to lipid accumulation and renal lipotoxicity [64,65]. It is known that intracellular lipid deposition is increased in humans and mouse models of tubulointerstitial fibrosis, while genes associated with fatty acid oxidation and OXPHOS, such as PPARα, PPARγ, CPT1, and Aconitase 2 (ACO2), are decreased [66,67]. Lipotoxicity may be either a cause or a consequence of mitochondrial dysfunction [68]. Abnormal fatty acid metabolism and mitochondrial dysfunction contribute to renal pathology by promoting inflammation, oxidative stress, and fibrosis [62].

Except for reduced lipid consumption, increased lipid uptake and fatty acid synthesis as well as decreased lipid export also contribute to the lipid accumulation in the kidney [69]. CD36, also known as scavenger receptor B2, which has been identified as a long-chain FFA transporter and signal transduction molecule, mediates the binding and cellular uptake of long-chain fatty acids and oxidized lipids and phospholipids [70]. In the kidney, CD36 is mainly expressed in tubular epithelial cells, podocytes, and mesangial cells [69,71]. It has been reported that CD36 is markedly upregulated in renal diseases and plays a key role in fatty acid oxidation, lipid accumulation, oxidative stress, inflammatory signaling, energy reprogramming, apoptosis, and renal fibrosis [63,69,71,72]. Additionally, fatty acid transport proteins (FATPs), specifically FATP1, FATP2, and FATP4, which facilitate cellular uptake and synthesis of FFA, are also highly expressed in the kidney and may contribute to lipid accumulation and progression of CKD [63,73,74]. Moreover, lipid accumulation is also caused by deceased cholesterol efflux mediated by ATP-binding cassette transporter A1 (ABCA1) and G1 (ABCG1) and elevated cholesterol influx through the low-density lipoprotein receptor (LDLR) [75].

**Figure 1:**
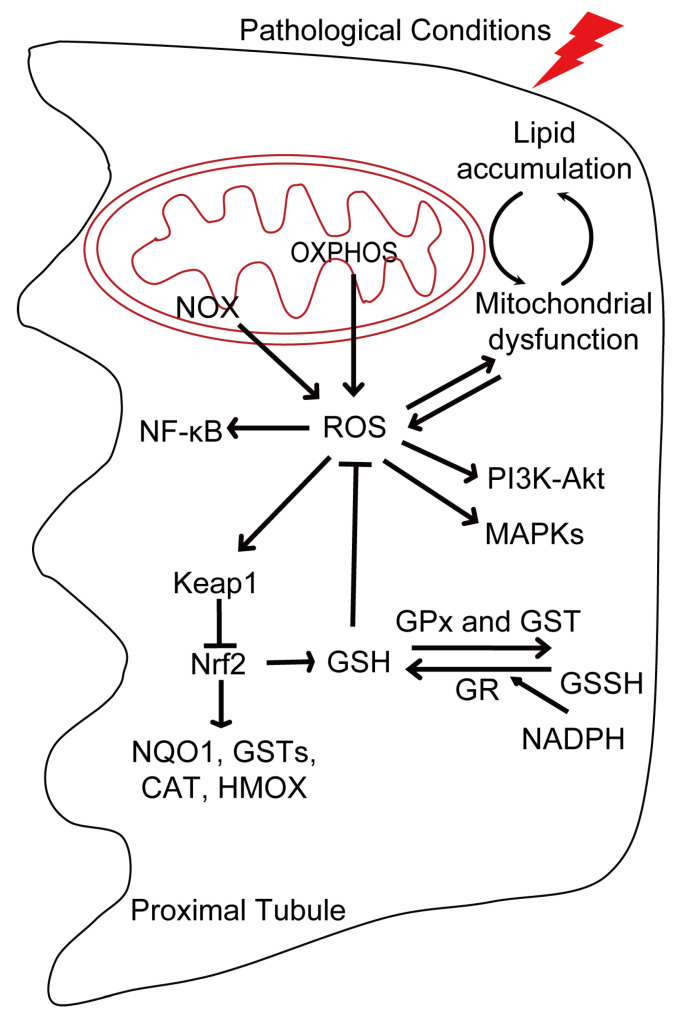
Schematic representation of the interplay between the redox system and mitochondrial metabolism. Mitochondria and the nicotinamide adenine dinucleotide phosphate oxidase (NOX) family are the major sources of endogenous ROS. Oxidative phosphorylation (OXPHOS) leads to the generation of ROS as metabolic by-products. Under pathological conditions, the interplay between lipid accumulation and mitochondrial dysfunction leads to ROS production. In turn, increased ROS lead to mitochondrial dysfunction and lipid accumulation. ROS play a critical role in Nuclear Factor kappa B (NF-κB), mitogen-activated protein kinases (MAPKs), phosphatidylinositol-3-kinase (PI3K)-Akt, and the KEAP1-NRF2-ARE signaling pathways. ROS are counteracted by antioxidants such as Nrf2 and GSH. Nrf2 upregulates target genes such as NAD(P)H quinone dehydrogenase 1 (NQO1), glutathione S-transferases (GSTs), catalase (CAT), and heme oxygenase 1 (HMOX), which play a critical role in mitigating oxidative stress, participating in detoxification pathways and increasing glutathione synthesis. The GSH pool is regulated by GPx and GST, which convert GSH to oxidized glutathione (GSSG); GR then reduces GSSG to GSH at the expense of NADPH, restoring the cellular GSH pool.

Unlike proximal tubule, distal tubular segments and glomerular cells, including podocytes, endothelial cells, and mesangial cells, are highly dependent on glucose to produce ATP for basal cell processes, with FA being used as an alternative substrate [4,49]. However, in renal diseases such as DKD, due to the high filtration rate, more ATP and oxygen consumption are required, resulting in a switch of metabolic fuel sources from glucose to fatty acids, contributing to renal hypoxia and increased oxidative stress [57]. The interplay between the redox system and mitochondrial metabolism is summarized in Figure 1.

## 4. Key Molecules Associated with Renal Tubular Transport and ROS

### 4.1. Transporters for Waste Removal

The kidney plays a key role in the excretion of waste products of both endogenous and exogenous origin and excess fluid through the urine to maintain the internal homeostasis. Except for glomerular filtration, tubular excretion and reabsorption are the main processes that determine renal function. These processes depend mainly on the transporters expressed on the basolateral and apical membranes of the proximal, distal, and collecting tubule epithelia [76]. Increasing evidence suggests that CKD may affect expression and function of membrane transporters in the kidney [76]. Dysfunction of transporters leads to deficient elimination of xenobiotics and endogenous byproducts and subsequent clinical consequences [76,77]. Membrane transporters are divided into two major superfamilies, solute carrier (SLC) and ATP-binding cassette (ABC) transporters [76]. The most abundant and predominant renal transporters include SLC transporters—organic anion transporter 1 (OAT1), 3 (OAT3), organic cation transporter 2 (OCT2), multidrug and toxin extrusion protein 1 (MATE1), as well as ABC transporters—P-glycoprotein (P-gp), multidrug resistance protein 2 (MRP2), and multidrug resistance protein 4 (MRP4) [78,79]. These transporters engage in the handling of creatinine, uremic toxins, and drugs in the kidney [80,81,82,83]. Additionally, OAT1 and OAT3 also mediate the uptake of uric acid from blood into tubular cells, and urate transporter 1 (URAT1) and OAT4 are major urate reabsorption transporters, while MRP4 and breast cancer resistance protein (BCRP) participate in the excretion of uric acid from tubular cells to the nephron lumen [84,85,86]. Uric acid has both antioxidant and pro-oxidant properties in vitro. Specifically, the circulating uric acid shows the antioxidant potential in human blood. However, when uric acid is inside the cells, it exhibits a pro-oxidant behavior, stimulating the production of free radicals and pro-inflammatory cytokines, which may be associated with the pathogenesis and progression of CKD [87,88,89].

### 4.2. Nutrient Transporters

Apart from removing waste products from the blood, the kidneys reabsorb all filtered nutrients, maintain electrolyte and fluid balance and acid–base homeostasis, and regulate blood pressure. These functions, particularly the reabsorption of amino acids, glucose, ions, and vitamins, primarily rely on plasma membrane transporters in the proximal tubules. Amino acids are reabsorbed from the tubular lumen by sodium–amino acid cotransport driven by Na^+^ electrochemical gradient across the luminal membrane established by the Na^+^-K^+^-ATPase [90,91]. Glutamine is the most abundant amino acid and plays pleiotropic roles in biosynthesis, acid–base regulation, maintenance of redox balance, energy production, and signal transduction processes [91,92]. Glutamine reabsorption is guaranteed by the membrane transporters alanine-serine-cysteine transporter-2 (ASCT2) and BOAT1, while the transporters sodium-coupled neutral amino acid transporter-3 (SNAT3) and L-type amino acid transporter (LAT1) mediate the glutamine flux from blood to tubular cells [90,91]. Glutamine is converted to glutamate after influx into cells and used, together with cysteine and glycine, to generate GSH and maintain redox homeostasis [93,94].

Glucose reabsorption from glomerular filtration depends mainly on three membrane proteins in the proximal tubule: sodium-glucose co-transporter 2 (SGLT2) expressed in the apical membrane of the S1 and S2 segments, sodium-glucose co-transporter 1 (SGLT1) found in the apical membrane of the S3 segment, and glucose transporter 2 (GLUT2) located in the basolateral membrane of the S1/S2/S3 segments [95,96,97,98]. SGLT2 resorbs more than 90% of the glucose filtered by the glomerulus, and the remaining glucose is reabsorbed by SGLT1 [99,100]. GLUT2 mediates glucose transport across the basolateral membrane towards to the plasma [101]. In addition, sodium-glucose co-transporter 3 (SGLT3) may serve as a glucose sensor, and sodium-glucose co-transporter 4 (SGLT4) mainly acts as a mannose transporter [96,102]. Sodium-glucose co-transporter 5 (SGLT5) is the major luminal transporter responsible for renal fructose reabsorption [103]. Glucose transporter 1 (GLUT1) may contribute to glucose reabsorption from the peritubular space [96]. Glucose reabsorption by SGLT2 and SGLT1 is also achieved by Na^+^-K^+^-ATPase, which transports Na^+^ out of the cell and generates a negative interior voltage, thereby providing concentration gradients for Na^+^-coupled glucose uptake by SGLT2 and SGLT1 [96].

### 4.3. Ion Transporters

**Figure 2:**
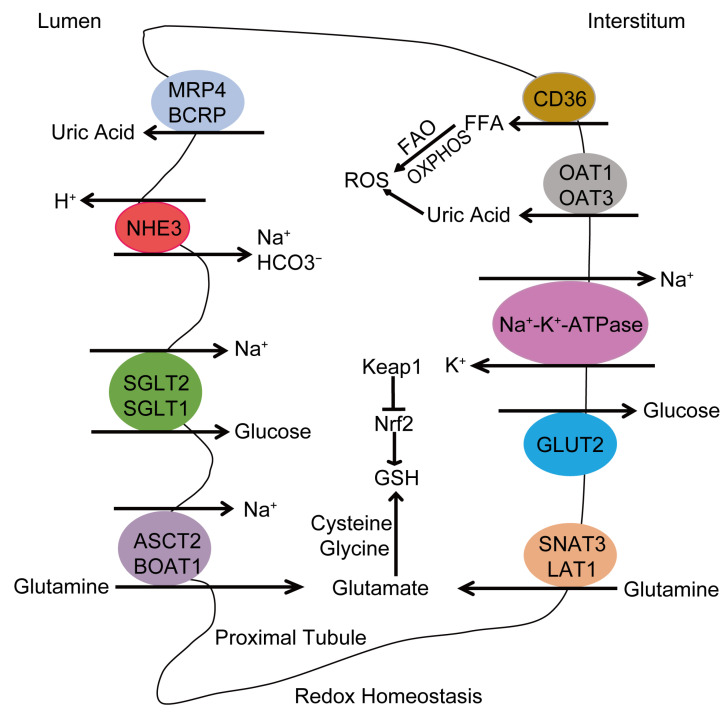
Schematic representation of key molecules involved in renal tubular transport and the redox system under physiological conditions. Under unstressed conditions, CD36 transports free fatty acids (FFAs) into the proximal tubular cells, which are then oxidized by fatty acid oxidation (FAO) and oxidative phosphorylation (OXPHOS) in the mitochondria. This process generates reactive oxygen species (ROS) as by-product. Organic anion transporter 1 (OAT1) and 3 (OAT3) mediate the uptake of uric acid from the blood into tubular cells, which exhibits a pro-oxidant behavior. MRP4 and breast cancer resistance protein (BCRP) are involved in the excretion of uric acid from tubular cells into the lumen of the nephron, thereby reducing intracellular levels of uric acid. Na^+^-K^+^-ATPase pumps 3 Na^+^ out of the cell and 2 K^+^ into the cell, creating an ion gradient across the cell membrane that drives the reabsorption of glucose and amino acids. Na^+^/H^+^ exchanger 3 (NHE3) reabsorbs HCO_3_^−^ and about 80% of Na^+^ and also secretes H^+^. Glucose reabsorption depends mainly on sodium-glucose co-transporter 2 (SGLT2) and sodium-glucose co-transporter 1 (SGLT1). Glucose transporter 2 (GLUT2) mediates glucose transport across the basolateral membrane towards to the plasma. Glutamine is the most abundant amino acid in tubular cells and is reabsorbed through alanine-serine-cysteine transporter-2 (ASCT2) and BOAT1. Sodium-coupled neutral amino acid transporter-3 (SNAT3) and L-type amino acid transporter (LAT1) mediate the glutamine flux from blood to tubular cells. Once inside the cells, glutamine is converted to glutamate and used, along with cysteine and glycine, to produce GSH. GSH is regulated by the master regulator of redox balance, Nrf2, and Nrf2 transcriptional activity is inhibited by its main inhibitor, Keap1.

Na^+^-K^+^-ATPase is located on the basolateral membrane throughout the nephron and is the main and most important transporter of Na^+^ [104]. Mitochondria provide the energy for Na^+^-K^+^-ATPase to pump 3 Na^+^ out of the cell and 2 K^+^ into the cell, creating ion gradients across the cell membrane [105]. This sodium gradient is also necessary for the kidney to filter waste products, regulate blood electrolyte levels, reabsorb amino acids, and maintain pH. Na^+^-K^+^-ATPase is critical for the maintenance of several cellular processes, and its dysfunction results in several pathological conditions [106,107]. Na^+^-K^+^-ATPase is a transmembrane protein composed of α, β, and γ subunits. The α-subunit is the catalytic domain and contains the binding site for Na^+^, K^+^, ATP, steroid hormones, and phosphorylation sites for protein kinase A (PKA) and protein kinase C (PKC) [108]. The β-subunit is involved in the structural and functional maturation of the enzyme [109,110]. The γ-subunit fine-tunes Na^+^-K^+^-ATPase in the kidney [108]. Na^+^-K^+^-ATPase is highly sensitive to alterations in redox state [111]. Specifically, increased ROS or RNS oxidize the Na^+^-K^+^-ATPase α/β subunits and its independent regulator FXYD proteins via S-glutathionylation, S-nitrosylation, phosphorylation, and carbonylation [106,111,112,113,114]. These oxidative modifications inhibit the Na^+^-K^+^-ATPase enzymatic activity and promote its degradation in renal proximal tubular cells [106,115]. But the oxidative modifications of Na^+^-K^+^-ATPase are reversible [116,117]. Moreover, Na^+^-K^+^-ATPase activity also regulates ROS production [106]. The major membrane transporters involved in renal tubular transport and the redox system under physiological conditions are summarized in Figure 2.

## 5. Membrane Transporters Regulate ROS Generation and Are Involved in Kidney Disease

ROS are necessary for physiological processes in the kidney, but loss of redox homeostasis contributes to proinflammatory and profibrotic pathways and proteinuria [24,38]. Energy metabolism and redox status are closely linked [118]. Metabolic pathways and antioxidant systems are coordinately regulated to maintain redox homeostasis. However, this balance is disturbed in kidney disease. DKD is the main cause of end-stage renal disease (ESRD) in developed countries, and oxidative stress (OS) is one of the main mechanisms of DKD [119]. Under high glucose conditions, cellular metabolism is switched from FAO to glycolysis, which promotes mitochondrial ROS production and stimulates CD36 expression in HK-2 cells. Inhibition of CD36 expression upregulates the level of FAO-related enzymes and significantly inhibits ROS production, protecting diabetic db/db mice from tubulointerstitial inflammation and tubular epithelial cell apoptosis [14].

Another membrane transporter that indirectly affects oxidative stress and is being investigated in patients with DKD is the ATP-binding cassette A1 (ABCA1). ABCA1 is involved in cholesterol and phospholipid efflux, which is significantly decreased in clinical and experimental DKD [120]. Selective induction of ABCA1 promotes the removal of excess cholesterol from podocytes, thereby stabilizing mitochondrial cardiolipin (a mitochondrial-specific phospholipid) in podocytes in DKD [121]. Additionally, in type 2 diabetic mice, ABCA1 deficiency in glomerular endothelial cells exacerbates glomerular cholesterol accumulation and glomerular endothelial injury and dysfunction such as increased creatinine levels, more severe proteinuria, mesangial matrix expansion and fusion of foot processes, and more pronounced renal inflammatory injury and cell death [15]. Conversely, ABCA1 overexpression enhances cholesterol efflux and significantly protects against glomerular endothelial injury in human renal glomerular endothelial cells (HRGECs) stimulated by high glucose and high cholesterol [15].

## 6. Redox Regulation in Kidney Disease

ROS have been shown to play a major role in the pathogenesis of renal inflammation, glomerular proteinuria, and fibrosis, subsequently contributing to hypertension, DKD, AKI, and CKD [6,11,122,123,124,125,126,127,128]. For example, catalase deficiency increases mitochondrial ROS and fibronectin expression in the development of diabetic mice [129]. Similarly, endothelial-specific deletion of the thioredoxin reductase 2 in mice leads to thickening of the Bowman’s capsule, glomerulosclerosis, and renal dysfunction [130]. Overexpression of glutathione peroxidase-1 reduces mitochondrial ROS and total cellular ROS and subsequently ameliorates age-related glomerulosclerosis, tubular atrophy, interstitial fibrosis, and loss of cortical mass in aged mice [131]. Hypertension and chronic kidney disease are intricate. Hypertension can worsen renal function, and progressive chronic kidney disease contributes to the exacerbation of hypertension. Catalase overexpression reduces ROS generation and pro-fibrotic and apoptotic gene expression in the renal proximal tubular cells, which prevented hypertension, albuminuria, tubulointerstitial fibrosis, and tubular apoptosis in the mouse model of hypertension [132].

One of the major chronic complications of diabetes is DKD. Increasing evidence shows that oxidative stress plays a critical role in the progression of DKD [133,134,135]. Nrf2 is abundantly expressed in diabetic kidneys. Endogenous antioxidants, through Nrf2, play a critical role in protecting the body from oxidative stress damage [37,46]. It has been reported that Nrf2 knockout in type I diabetic mice (Akita mice) results in an enlarged capillary loop in the glomeruli and distal tubules. Moreover, the expression of genes related to GSH synthesis is decreased in the kidney of Nrf2-knockout Akita mice [46]. Catalase overexpression in renal proximal tubule cells inhibits Nrf2 and heme oxygenase-1 (HO-1) gene expression, attenuates renal injury, and normalizes systolic blood pressure in type I diabetic mice (Akita mice) compared with controls [136]. However, the role of Nrf2 in DKD is controversial. It has been shown that global deletion of Nrf2 attenuates renal injury and tubulointerstitial fibrosis and reduces systolic blood pressure (SBP) in Akita mice [137].

Nrf2 has also been shown to protect the kidney from oxidative stress damage caused by ischemia-reperfusion injury (IRI)-induced AKI [138]. Specifically, tubular Nrf2 activation elevates gene expression of antioxidant and NADPH synthesis enzymes and ameliorates the progression of IRI-induced tubular damage [138]. Alteration of the redox state has also been observed in folic acid (FA)-induced AKI, which is thought to be the main mechanism responsible for renal injury [139]. Similarly, cisplatin induces ROS production and oxidative damage in the kidney, whereas restoration of redox balance protects renal cells from cisplatin-induced damage [140]. Furthermore, Nrf2 deficiency aggravates tubular damage, transdifferentiation, fibrosis, and inflammation while decreasing antioxidant responses after unilateral ureteral obstruction (UUO) [141].

## 7. Redox Regulation of Kidney Disease by Membrane Transporters

Increasing evidence shows that redox signaling regulates renal tubular transport by modulating the activity and expression of SGLT, basolateral Na^+^-K^+^-ATPase, and apical Na^+^/H^+^ exchanger 3 (NHE3), etc. Kidneys from diabetic patients exhibit higher levels of Nrf2 and SGLT2 in the renal proximal tubular cells than controls [99]. Oxidative stress induced by high glucose or exogenously added H_2_O_2_ inhibits the levels and activity of SGLTs and NHE3 in renal proximal tubule cells [9]. Overexpression of Nrf2 in renal proximal tubular cells results in increased blood glucose level, glomerular filtration rate, urinary albumin to creatinine ratio, tubulointerstitial fibrosis, and increased SGLT2 expression in Akita mice [99]. Similarly, in type 2 diabetic (T2D) db/db mice, deletion of Nrf2 decreases the fasting blood glucose, renal hypertrophy, glomerular filtration rate, urinary albumin/creatinine ratio, tubular lipid droplet accumulation, and systolic blood pressure, via down-regulation of SGLT2, CD36, and FABP4 expression in proximal tubular cells [44]. The reduced expression and activity of SGLT2 may explain the increased transforming growth factor-beta1 secretion and the activation of the NF-κB signal transduction pathway [127,142]. Moreover, a global decrease in sodium transporters including NHE3, sodium chloride cotransporter (NCC), Na-K-2Cl cotransporter (NKCC2), and epithelial sodium channel (ENaC) has been observed in the kidney of UUO models [143].

Metabolic acidosis increases Nrf2 activity and expression of the glutamine transporter Slc38a3 (SNAT3) in the kidney. Nrf2 knockout fails to induce Snat3 expression during metabolic acidosis and shows significantly increased expression of renal markers of oxidative stress and injury, indicating that NRF2 regulates the SNAT3 in response to metabolic acidosis [10]. Drug transporters are protein pumps that involved in the efflux and influx of endogenous molecules and xenobiotics using ATP hydrolysis or ion/concentration gradients [144]. Knockdown of Keap1 in human tubular epithelial cells elevates the expression of four renal transporters—multidrug resistance protein 1 (MDR1; ABCB1), breast cancer resistance protein (BCRP; ABCG2), multidrug resistance-associated protein 2 (MRP2; ABCC2), and MRP3 (ABCC3)—at both gene and protein levels [145].

**Figure 3:**
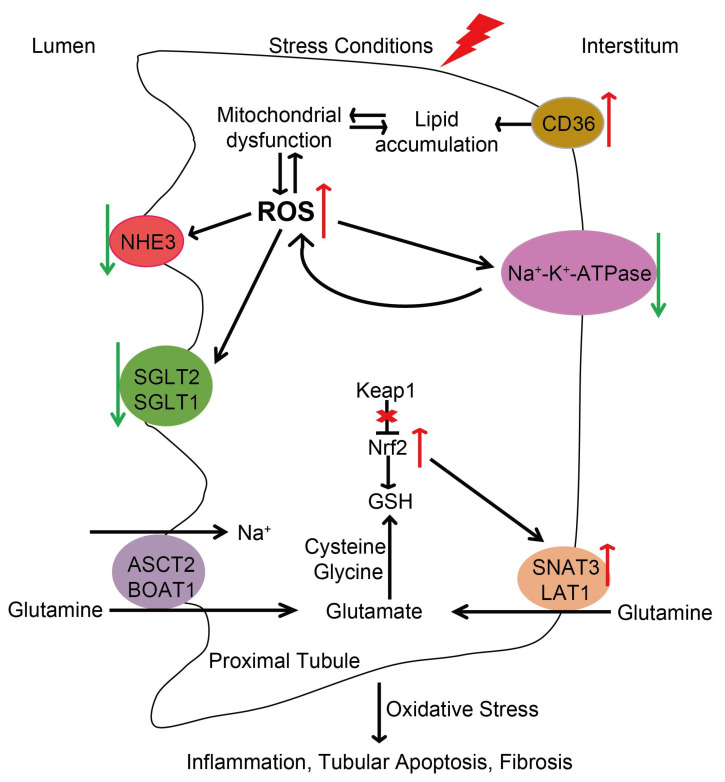
Schematic representation the interplay between key transporters and the redox system under stress conditions. Under stress conditions, CD36 expression is increased and transports more FFA into proximal tubular cells, leading to lipid accumulation and subsequent mitochondrial dysfunction and ROS production. In turn, increased ROS reduce mitochondrial lipid utilization, resulting in lipid accumulation and renal lipotoxicity. Increased ROS oxidize Na^+^-K^+^-ATPase subunits and promote Na^+^-K^+^-ATPase degradation, which may affect glucose and glutamine reabsorption. However, ROS activate Na^+^-K^+^-ATPase signaling pathways, which further increases the generation of mitochondrial ROS generation through the positive-feedback oxidant amplification loop. Oxidative stress inhibits the levels and activity of SGLTs and NHE3 in renal proximal tubule cells. Nrf2 is released from Keap1 and induces the expression of antioxidant genes. Nrf2 upregulates the expression of the glutamine transporter SNAT3 during metabolic acidosis. The effect of Nrf2 on SGLT2 is controversial. Overall, oxidative stress leads to renal inflammation, tubular apoptosis, and fibrosis and contributes to the development and progression of kidney disease. The lightning symbol indicates under stress conditions. The red arrows indicate increased expression, while the green arrows indicate decreased expression. Red “X” indicates prevention of the effect of Keap1 on Nrf2.

Nutrient reabsorption is highly dependent on membrane transporters driven by Na^+^-K^+^-ATPase. Na^+^-K^+^-ATPase is redox-sensitive and may mediate the interplay between ROS and nutrient reabsorption in proximal tubular cells. Increased ROS oxidize the Na^+^-K^+^-ATPase α/β subunits and its independent regulator FXYD proteins, subsequently inhibiting its activity and promoting its susceptibility to degradation by proteasomal and endosomal/lysosomal proteolytic pathways [115]. Furthermore, in addition to regulating renal sodium handling, Na^+^-K^+^-ATPase plays a critical role in Src-mediated signal transduction, leading to the activation of downstream key signaling pathways (such as epidermal growth factor receptor, PI3K, Ras/Raf/ERK, PLC/PKC, and p42/44 mitogen-activated protein kinases) and the initiation of kinase cascade signaling, which plays an important role in the regulation of cell proliferation, differentiation, and apoptosis [11,146]. Stimulation by specific ligands, i.e., cardiotonic steroids (CTS), or ROS activates Na^+^-K^+^-ATPase signaling pathways, which further increases the generation of mitochondrial ROS generation through the positive-feedback oxidant amplification loop [116,147,148]. Na^+^-K^+^-ATPase signaling has been implicated in inflammation, oxidative stress, renal fibrosis, and renal proximal tubule sodium reabsorption [148,149,150]. For example, H_2_O_2_ modestly reduces the number of Na^+^-K^+^-ATPase in the surface of LLC-PK1 cells and decreases the expression of E-cadherin through direct activation of the Na^+^-K^+^-ATPase/Src-mediated signaling pathway [11]. Intracellular H2O2 also regulates the Na^+^-K^+^-ATPase expression and activity in cultured opossum kidney (OK) cells [12]. Inhibition of Na^+^-K^+^-ATPase significantly increases the protein expression and nuclear localization of the transcription factor Snail in the tubular epithelia [151]. Moreover, Na^+^-K^+^-ATPase has been implicated in the generation of ROS and the development of anemia in CKD [107]. NHE3 is located in the apical membrane of the S1 and S2 segments, which mediate transcellular reabsorption of Na^+^ and HCO_3_^−^ and secretion of hydrogen [152]. Approximately 80% of the Na^+^ was reabsorbed by the Na^+^/H^+^ exchanger (NHE) [13]. NADPH oxidase-derived ROS reduce Na^+^ transporters activity and fluid reabsorption, which may be mediated by NHE3 in the proximal tubule [125,128]. The NKCC2 is located in the apical membrane of the epithelial cells of the thick ascending limb of Henle’s loop (TAL) [153,154]. Furthermore, 20–25% of the total filtered NaCl was shown to be reabsorbed by NKCC2 [155,156]. The luminal flow-stimulated increase in NKCC2 activity in the thick ascending limb is mediated by NADPH oxidase 4-derived superoxide, which enhances NaCl reabsorption in the thick ascending limb [157]. Additionally, Nrf2 regulates the expression of the NCC [158]. The interplay between key transporters and the redox system under stress conditions is summarized in Figure 3.

## 8. Conclusions and Perspectives

Redox homeostasis is essential for a wide range of cellular processes and human health. Redox state and energy metabolism, especially lipid metabolism, are tightly coupled in the kidney. Cellular metabolic dysfunction is widely observed in various renal diseases and is probably the main source of ROS. Redox imbalance leads to abnormal renal function and contributes to the development and progression of several serious renal diseases. Although many antioxidant molecules have shown therapeutic potential in preclinical studies, the results of clinical trials have been disappointing. Therefore, a comprehensive understanding of redox regulation under physiological and pathological conditions will facilitate the development of redox medicine and benefit patients with redox-associated kidney diseases. Here, we explain that redox regulates the expression and function of key molecules involved in renal tubular transport and that dysfunction of membrane transporters in turn leads to inadequate excretion of wastes and reabsorption of nutrients with subsequent redox imbalance and clinical consequences. We hope that a comprehensive study of the interplay between the redox system and membrane transporters in the kidney will provide a rational approach that will promote precision medicine and lead to greater pharmacological success.

## Data Availability

Not applicable.
